# Early Clinical Exposure in Pre-clinical Years of Medical School

**DOI:** 10.31729/jnma.5341

**Published:** 2021-10-31

**Authors:** Anisha Basukala, Kabita Chaudhary

**Affiliations:** 1 Nepalese Army Institute of Health Sciences, Sanobharyang, Kathmandu, Nepal

**Keywords:** *clinical skills*, *medical education*, *self-directed learning*

## Abstract

Medical science is one of the sectors which has faced rapid advancement in the past few years. But sadly, medical schools are still following the traditional curriculum where a wide gap between the pre-clinical and clinical phases prevails. Early clinical exposure is known to act as a bridge to this gap. It contributes to the overall development of naive medical students enhancing their communication skills, clinical skills, teamwork, empathy development, and motivation towards selfdirected learning and hence making them more competent for clinical years. So, as it reinforces the conventional didactic teaching-learning method it becomes a much-needed part for the students in preclinical years.

## INTRODUCTION

The medical education curriculum was divided into preclinical and clinical phases by Abraham Flexner back in 1910.^1^ This system has been implemented all around the world including Nepal. There has been rapid advancement in medicine since then. But, the problem at present is that “Physicians of tomorrow are taught by teachers of today using curricula of yesterday".^2^ This has led experts to feel the need for modifications to this system.

In Nepal, under Tribhuvan University (TU) the curriculum of Bachelors of Medicine and Bachelors of Surgery (MBBS) is divided into two years of the preclinical phase and two and half years of the clinical phase.^3^ The preclinical years include a didactic method of teaching-learning where students are taught the basic science topics (Anatomy, Physiology, Pharmacology, Pathology, Biochemistry, and Microbiology) and Community Medicine. This basic phase has been isolated from clinical training in the traditional format. ECE acts as a bridge that fills the profound gap between preclinical and clinical phases and minimizes the line of demarcation between them. To address this, Tribhuvan University-Institute of Medicine (TU-IOM) included early clinical exposure (ECE) in the curriculum, revised in 2008.^3^ This curriculum has followed the approach of history taking, communication skills, problem-based learning, and community health diagnosis to promote early clinical exposure.^3^ However, the methods for teaching/learning and assessment are not well defined in the curriculum.

There is no concrete definition of ECE in the literature, but it can be defined as 'a teaching-learning methodology, which fosters exposure of the medical students to the patients as early as the first year of a medical college in a social or clinical context that enhances the learning of health, illness or disease, and the role of the health professional.'^1^ The three main elements of ECE are: basic science correlation, clinical skills and humanities in medicine.^4^ It can be carried out in three settings which include classroom setting, hospital setting, and community setting.^1^ In classroom setting, cooperative and uncomplicated cases can be brought for case discussion and if a patient cannot be brought, a paper-based case can be used as a trigger for discussion.^1^ In hospital settings, students are taken to ward-visits, made to understand the protocols, and taught basics about history taking. In community settings, students are enabled to interact with a community which makes them aware of how people live, how their living conditions influence their health, and their knowledge, attitude, and practice towards health-related conditions. Through these settings, ECE aims to provide a better conceptualization of basic science topics and supplement the effect of conventional teaching methods. So, the purpose of ECE is not to replace the conventional didactic lectures nor to prepone the clinical teaching but to reinforce the concepts of basic sciences through a clinical context.

The benefits of ECE for students are mainly reported in the following areas:

## 1. COMMUNICATION SKILL

The introduction of clinical exposure during preclinical years has shown to help the majority of medical students develop good communication skills.^5^ Good communication skill is a boon not only when dealing with patients but also with peers and in clerical tasks. In the medical profession, better communication between doctor and patient builds confidence, improves compliance, and thereby reduces mistakes and mishaps and, prevents malpractice suits.^6^

## 2. MOTIVATION TOWARDS SELF-DIRECTED LEARNING

In the didactic method of teaching, students are taught in a classroom without any clinical exposure where students feel difficult to correlate the concepts. Early clinical exposure helps students to correlate better with vertical and horizontal integration.^7^ This has shown to improve students' motivation to learn more and also improve retention.^8^ Motivation towards self-directed learning elevates the student's confidence about what they are learning. Early clinical exposure has been found to have enhanced the performances of students in Objective Structured Clinical Examination (OSCE) and university exams.^9^,^10^

## 3. LEARNING BY DOING

Benjamin Franklin quotes, “tell me and I forget, teach me and I may remember, involve me and I learn." Early clinical exposure provides opportunities for medical students to learn clinical skills and actual patient handling from the very first year. It has the potential to improve the cognitive, psychomotor as well as affective domains of medical students which is essential for inculcating professional attributes into their behaviors.^7^,^11^

## 4. EMPATHY

Dealing with emotions is an important feature of professional behavior in medicine. Empathy leads to better patient cooperation and compliance which results in better outcomes. ECE fosters emotional development in medical students through active participation in medical practice and patient interaction as reported in studies where students were involved in the early clinical courses.^12,13^

## WORKING IN A TEAM

As students are allowed to work in groups as in problem-based learning, case-based learning they get opportunities to know each other better. With the understanding of group dynamics, they can move ahead as a team both inside and outside the medical school. This helps in improving their listening skills and problem-solving skills and building leadership skills as they relate to an interdisciplinary team.^1^ Furthermore, it also contributes to enhance critical thinking and analysis which helps in the reduction of errors in their work.

Personally, we believe that early clinical exposure is highly important in the present context where the world has grown more competent and advanced than ever. During our preclinical years, we were involved in ward-visits, history taking, communication skills learning sessions, community health diagnosis, problem-based learning, correlation seminars, and cadaver-based workshop on abdominal paracentesis. Some of these were part of our curriculum and some were added by our teachers so that we get early exposure to clinical settings. We are very grateful to them for these opportunities. These activities have contributed a lot to our overall development making us more confident and responsible during our clinical years. We learned how to initiate communication and build a good rapport with patients and patients' parties. We learned to work in a team supporting each other and growing together. This exposure motivated us to pursue our dream of wearing a white coat and Littmann around the neck with dignity so, we found ourselves more focused on our studies.

## WAYS FORWARD

Early clinical exposure is found to be beneficial wherever it has been practiced. Therefore experts from different countries have recommended the inclusion of Early clinical exposure in the medical curriculum including Nepal and India.^14,15^ But it is not free of limitations. It requires more resources, more time, more energy, proper training, and coordination among the faculties which can be overcome with the joint effort of faculties and students. Considering the fact that its necessities outweigh its limitations, it is high time that the 'curriculum of yesterday' be modified to teach the 'physicians of tomorrow.'
